# From hypertension control to global cardiovascular risk management: an educational intervention in a cluster-randomised controlled trial

**DOI:** 10.1186/s12875-015-0274-1

**Published:** 2015-05-07

**Authors:** Achim Mortsiefer, Tobias Meysen, Martin Schumacher, Heinz-Harald Abholz, Karl Wegscheider, Jürgen in der Schmitten

**Affiliations:** Institute of General Medicine, Medical Faculty, Heinrich-Heine-University Düsseldorf, P.O. Box 101001, 40225 Düsseldorf, Germany; Department of Medical Biometry and Epidemiology, University Medical Center Hamburg-Eppendorf, Martinistrasse 52, 20246 Hamburg, Germany

**Keywords:** Hypertension, Cardiovascular diseases, Prevention and control, Primary care, Educational intervention

## Abstract

**Background:**

Guidelines on hypertension management recommend adjusting therapeutic efforts in accordance with global cardiovascular risk (CVR) rather than by blood pressure levels alone. However, this paradigm change has not yet arrived in German General Practice. We have evaluated the effect of an educational outreach visit with general practitioners (GPs), encouraging them to consider CVR in treatment decisions for patients with hypertension.

**Methods:**

Prospective cluster-randomised trial comprising 3443 patients with known hypertension treated by 87 GPs. Practices were randomly assigned to complex (A) or simple (B) intervention. Both groups received a guideline by mail; group A also received complex peer intervention promoting the concept of global CVR. Clinical data were collected at baseline and 6-9 months after intervention. Main outcome was improvement of calculated CVR in the predefined subpopulation of patients with a high CVR (10-year mortality ≥5%), but no manifest cardiovascular disease.

**Results:**

Adjusted for baseline the follow-up CVR were 13.1% (95% CI 12.6%-13.6%) (A) and 12.6% (95% CI 12.2%-13.1%) (B) with a group difference (A vs. B) of 0.5% (-0.2%-1.1%), p = 0.179. The group difference was -0.05% in patients of GPs familiar with global CVR and 1.1% in patients of GPs not familiar with with global CVR. However, this effect modification was not significant (p = 0.165). Pooled over groups, the absolute CVR reduction from baseline was 1.0%, p < 0.001. The ICC was 0.026 (p = 0.002). Hypertension control (BP <140/90 mmHg) improved in the same subpopulation from 38.1 to 45.9% in the complex intervention group, and from 35.6 to 46.5% in the simple intervention group, with adjusted follow-up control rates of 46.7% (95% CI 40.4%-53.1%) (A) and 46.9% (95% CI 40.3%-53.5% (B) and an adjusted odds ratio (A vs B) of 0.99 (95% CI 0.68-1.45), p = 0.966.

**Conclusions:**

Our complex educational intervention, including a clinical outreach visit, had no significant effect on CVR of patients with known hypertension at high risk compared to a simple postal intervention.

**Trial registration:**

ISRCTN44478543.

## Background

Arterial hypertension is an important risk factor for the occurrence of cardiovascular disease (CVD). However, recent scientific understanding [1-4] and subsequently the published guidelines [5-8] recommend that the intensity of therapeutic efforts should no longer follow set limit levels, such as “<140/90 mm Hg”, but take individual CVR into account: “treat risk, not risk factors” [2].

This paradigm change, although receiving broad scientific consensus, has not fully reached health services research and clinical practice. Many surveys continue to report “hypertension control” rates without considering CVR [8,9], and the new concept remains unfamiliar in family practice [10,11]. Hypertension control in the relevant target group with manifest CVD or otherwise high CVR remains poor [12], despite improvements in some countries [13,14]. A recent Cochrane review on interventions used to improve control of blood pressure in patients with hypertension found that “none of the included RCTs attempted to manage hypertension in the context of overall cardiovascular risk”, concluding that “future studies need to be congruent with hypertension guidelines that recommend treatment and control of blood pressure in combination with multi-factorial risk reduction” [15].

If physicians are to consider global CVR before recommending anti-hypertensive treatment, they need to assess and discuss it with their patients. The use of CVR calculators in the consultation may improve patient satisfaction and involvement [16-18], although few physicians seem to use them [19]. However, little is known about the effects of this “double paradigm shift” [16] toward global CVR and shared decision making on clinical outcomes. Only a few intervention studies addressing hypertension management in primary care with explicit consideration of global cardiovascular risk have been published [20], of which only one measured CVR before and after the intervention [21].

The trial reported here evaluated the effect of a complex intervention in German General Practice, including an educational outreach visit, on global CVR of patients with known hypertension, compared to a simple intervention by mail.

## Methods

### Study design

We report a prospective non-blinded longitudinal cluster-randomised intervention trial involving general practitioners and their patients with known hypertension. The trial tested the superiority of a complex (A) over a simple (B) intervention at the physician (cluster) level, whereas outcomes were measured as improvement of CVR at patient level.

The detailed protocol of CRISTOPH (cluster-randomised intervention study to optimise the treatment of patients with hypertension) has already been published [22], as has a secondary analysis of the baseline data [23]. The trial was registered at ISRCTN44478543.

### Practices and participants

89 general practitioners (GPs) were recruited in 3 regions close to the cities of Düsseldorf, Cologne and Aachen. Each GP was asked to enrol a consecutive sample of 40 patients with a known diagnosis of hypertension from his or her daily patient flow, regardless of the current cause for consultation. Other inclusion criteria were age (40-75 years) and continuity of care by the GP over at least 6 months. Emergency cases and patients expected to die within one year were excluded.

Ethical approval (No. 2715) was obtained from the Ethics Committee of the medical faculty of the University of Düsseldorf. Patient consent was deemed unnecessary by the Committee since we use anonymous data routinely collected by GPs.

### Cluster randomisation

After enrolling the patients and recording of baseline data, GPs were cluster-randomised into 2 groups, A (complex intervention) and B (simple intervention). One GP (or one group practice with 1-3 GPs) represents a cluster, whereas the patients are the observation units.

To avoid the possibility that GPs familiar with the concept of global CVR were accidentally over-represented in either group, we asked them to estimate the CVR of each patient enrolled. This estimate was compared with the respective patients’ calculated CVR, and the GPs were divided into 2 groups according to whether their estimate was close to or far from the calculated CVR. ‘Close to’ was assessed if the mean deviation of the estimated from the calculated CVR was below 0.5% and the mean absolute deviation was <0.75% (both values were near the median for all GPs). Thus, 45 GPs were defined as familiar and 44 as not familiar with global CVR. The resulting strata were used for a stratified randomization to balance the familiarity between random groups. Because of the necessity to randomise all GP’s working in group practices into the same study group (A or B), stratified randomisation resulted in a higher imbalance (47 in A and 42 in B) than the otherwise expected 45 versus 44.

### Baseline data and subgroups by CVR

A total of 3,523 patients were enrolled, the baseline data being given in Table 1. There was no significant difference between groups A and B at baseline. 23% of all patients were reported to have a manifest cardiovascular disease (CVD), 47.7% had a high CVR (SCORE ≥5%) without manifest CVD, and 29.3% had a low CVR, defined by a cardiovascular 10-year mortality (SCORE) of <5%, without manifest CVD.

**Table 1:** **Baseline information**

	**Complex intervention (A)**	**Simplex intervention (B)**
**General practitioners**		
**No**	**47**	**42**
**Patients**		
No	1849	1674
Mean age (years)	63.8 ± 9.3	63.4 ± 9.1
No (%) female sex	993/1846 (53.8)	857/1672 (51.3)
No (%) of smokers	385/1809 (21.3)	300/1645 (18.2)
Mean BP systolic (mmHg)	137.8 ± 17.0	138.7 ± 16.9
Mean BP diastolic (mmHg)	81.5 ± 9.6	82.3 ± 9.2
Mean Total cholesterol (mg/dl)	216.0 ± 43.0	219.4 ± 44.8
No (%) with diabetes mellitus	623/1845 (33.8)	485/1671 (29.0)
No (%) renal failure	73/1841 (4.0)	56/1670 (3.4)
Mean 10-year risk of cardiovascular mortality	0.104 (±0.094)	0.099 (±0.088)
No (%) with manifest CVD (secondary prevention)	430/1755 (24.5)	340/1600 (21.3)
No (%) with ischemic heart disease	286/1845 (15.5)	236/1673 (14.1)
No (%) with cerebrovascular disease	107/1840 (5.8)	99/1672 (5.9)
No (%) peripheral artery disease	122/1841 (6.6)	87/1669 (5.29)
No (%) with no CVD (primary prevention)	1325/1755 (75.5)	1260/1600 (78.8)
No (%) with low CVR (10 year risk of cardiovascular mortality < 5%)	495/1755 (28.2)	488/1600 (30.5)
No (%) with high CVR (10 year risk of cardiovascular mortality ≥ 5%)	830/1755 (47.3)	772/1600 (48.3)

Quantitative values are expressed as the mean ± SD.

### Intervention

Implementation of clinical guidelines into professional practice presents a major challenge [24]. While it is difficult to change professional behaviour overall, with only small changes to be expected, complex interventions including educational outreach visits may yield a sustainable and relevant effect than simple measures, such as merely disseminating guidelines [25].

The assumption we made was that improvements in the GPs’ knowledge about the concept of global CVR and case-based training of their competence in using cardiovascular risk tables would lead to improvement in their management of CRV factors, particularly in patients at high risk.

#### Simple intervention

All participating physicians received by mail a written manual (17 pages) we had specifically developed for GPs on the basis of the ESC-Guidelines [6], and a set of 50 copies of the SCORE CVR tables, with a specifically designed patient information text overleaf. There was an accompanying letter encouraging the GPs to use these materials for the management of their patients with arterial hypertension.

Our concern was that the benefit of the complex, and therefore more costly, intervention should be regarded as relevant only if the effect could be demonstrated against a less complex cheaper intervention. Since passive dissemination of guidelines has had little or no effect on relevant clinical outcomes [26], this simple intervention probably constituted a factual “usual care” control (B) group.

#### Complex intervention

For the GPs assigned to the complex (A) intervention group, multi-faceted intervention was developed that included, in addition to the written materials disseminated by mail (cf simple intervention), a personal intervention comprising an educational outreach visit by a peer (30-45 min) and a feedback telephone call by the same peer 3 weeks later (5-10 min).

During the outreach visit, a peer and a GP discussed the new concept on the basis of: (a) the mailed manual, and (b) 3-4 suitable cases selected by the former from this GP’s baseline data. The cases had to include at least one patient with a history of CVD, and at least 2 patients without such a history. Of the latter, one would be at low risk (SCORE <5%), and one at high risk (≥5%). Discussion of the GPs’ respective patient cases included feedback elements, which served as concrete examples to demonstrate the paradigmatic changes of the CVR concept to the GP. The GPs were also instructed how to use the SCORE calculator of global CVR (print version), and were demonstrated the patient information leaflet.

Three practising GPs (TM, AM, and JidS) were trained to explore the GPs’ understanding, beliefs and attitudes during the initial phase of the conversation in order to tailor the concept’s message to him or her. Standardisation was facilitated by a dialogue draft for the outreach visit, regular audit-meetings, and personal feedback from a passive observer who attended the first 3 visits of each peer. The complex intervention (A) included the following elements:

- Provision of a written manual (17 pages) and patient information leaflets sent by mail to the GP (similar to the simple intervention B).
- Educational outreach visit of the GPs by peers, including instruction on how to use the SCORE CVR calculator.
- Feedback telephone call 3-4 weeks after the outreach visit.

The educational intervention was based on five key messages:

1. Therapeutic decisions in the management of hypertension should always be preceded by an estimation of absolute CVR.
2. Within certain limits, there are no fixed targets for blood pressure or cholesterol level. Potential risk reductions by drug intake or life-style modification depend on the absolute CVR before treatment.
3. All patients with hypertension should be encouraged to improve or continue a healthy lifestyle. Drug therapy is regarded beneficial in patients at high CVR (SCORE ≥5%) or with manifest CVD.
4. There are often several options to reduce CVR. If one option is barred (for example, because of adverse patient preferences or drug side effects), other options need to be considered.
5. Explaining individual CVR may enhance communication between the physician and the patient, providing the opportunity to invite the patient to share in decision-making.

The GPs were not informed of the existence of 2 different interventions, leaving them unaware of which intervention group they belonged.

### Data collection

Data collection procedures were the same in both groups. We employed a self-developed paper documentation because of the limited extent and availability of routine computerised data. At baseline, besides the sociodemographic and history data given in Table 1 the actual blood pressure reading (at enrolment) and the latest cholesterol measurement (before enrolment) were noted.

This study was designed with one follow-up data collection 6 to 9 months after the intervention that had been conducted during a routine visit of the patient in the study practice. Where available, the actual levels of blood pressure and cholesterol were recorded by a practice. New onset of diabetes mellitus, nephropathy or cardiovascular events was also recorded by the GPs, and smoking status updated.

### Time frame and participant flow

Between January and August 2006, 89 GPs were recruited, allowing the inclusion of 3,523 patients into the study. After finishing the baseline data collection, 47 GP’s were randomised to the complex intervention group A and 42 to the simple intervention group B.

**Figure 1:**
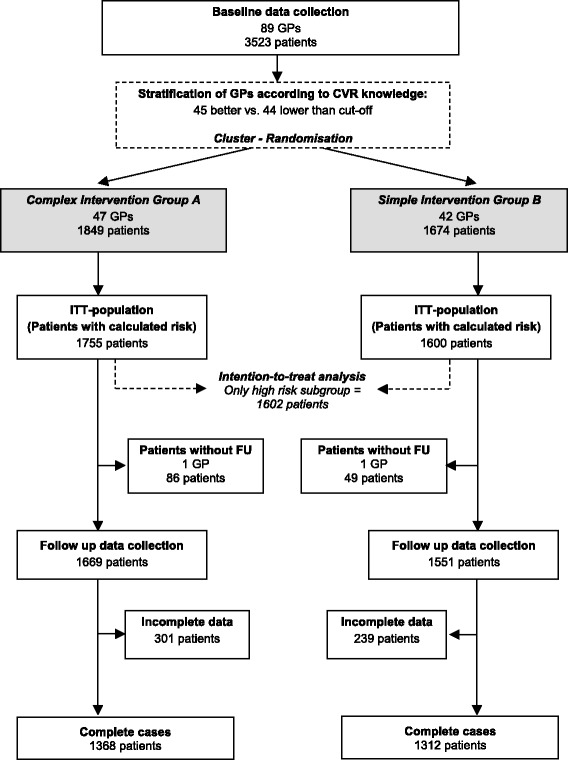
Study flow chart.

During the intervention period (from June 2006 to January 2007), 2 GPs and their corresponding study patients dropped out of the study. In the follow-up data collection (from January to November 2007), the remaining 87 GPs had documented 3,443 patients. In 2,680 cases, full data sets were available in order to compute 10-year CVR before and after intervention according to the SCORE formula. Practice and participant flow is given in Figure 1 as the “study flow chart”.

### Outcomes

We analysed changes in CVR, mean BP, total cholesterol level, smoking rate hypertension control rate, and CVR-lowering drug (ASS and statins). These were calculated separately for 3 different subpopulations of patients:

1. Patients with a history of manifest cardiovascular disease (CVD).
2. Patients with high CVR (SCORE ≥5%), but no history of CVD.
3. Patients with low CVR (SCORE < 5%), and no history of CVD.

Global CVR of the latter 2 subpopulations has been calculated employing the European SCORE formula, modified by factor 2 or 3 for men and women, respectively, with diabetes [6].

Primary outcome of this trial was a reduction of calculated CVR in the subpopulation of patients at high CVR (SCORE ≥5%) with no manifest CVD.

### Sample size calculation

To demonstrate an effect of reducing mean calculated CVR by 10% in the subgroup of patients with high CVR (SCORE ≥ 5%, no history of CVD) with a power of 80%, we calculated a necessary initial sample size of 2 × 51 recruited GPs each enrolling 40 patients. We estimated a GP drop out during the enrolment phase of 5%, a patient enrolment rate of 95%, a GP drop out between baseline and follow-up of a further 5%, and a patient drop out between baseline and follow-up of 10% (resulting in 3146 patients enrolled by 92 GPs). Sample size calculation was based on the assumption of a standard deviation of 0.44 for the CVR changes on the log-odds scale, an intra-class correlation of 0.2, and a proportion of 40% high risk patients in the total sample, the latter being derived from a pilot study of 330 patients cared for by 20 GPs. From these assumptions, 2 × 816 patients were required for the primary analysis in the high risk group.

### Statistical analysis

The analysis and the presentation of results follow the CONSORT guidance on cluster randomized trials [27]. Primary analysis included all randomized patients of the first (high CVR) subgroup with global CVR determinations at baseline (ITT population). In case of missing follow-up values, a LOCF (last-observation carried forward) was imputed, i.e. the baseline determination was imputed as follow-up determination. For the primary analysis, a 2-level random intercept model was fitted to the data with follow-up CVR as outcome, random group and CVR familiarity strata as fixed factors, baseline CVR as covariate and, with patients nested in physicians modeled as random effects. This hierarchical ANCOVA model takes the correlation structure resulting from cluster randomization into account and allows for differences between physicians in treatment effects. The primary analysis used the fixed effects contrast test that compares the mean follow-up CVR adjusted for baseline CVR between random groups. We report the estimated means with confidence limits, the group differences with confidence limits and p and the intraclass correlation coefficient (ICC) with corresponding p value. Effect modification by CVR familiarity of GP was tested by adding the corresponding interaction term to the hierarchical model. The reported p value results from the maximum likelihood test that compares the models with and without interaction term. If pre-post changes were to be analyzed subsequently, this was done for the random groups or for the total subpopulation with paired t tests for continuous outcomes or McNemar tests for binary outcomes.

As sensitivity analyses, calculations were repeated for the complete cases population and for a dataset with missing values imputed by an EM algorithm.

Secondary analyses involved only the complete cases. Analogous to the primary analysis, mixed models were fitted separately for the secondary endpoints in groups 1, 2 and 3. If the outcome was binary, the hierarchical ANCOVA model was replaced by a hierarchical logistic regression model with the corresponding factor, covariate and random part as in the ANCOVA models. As a post-hoc analysis, the pooled statin intervention effect was calculated across all types of patients.

Calculations used the last available versions of SPSS and STATA.

## Results

Baseline information is presented in Table 1. The outcomes of the study are reported separately for the 3 CVR subpopulations.

The primary analysis was in the ITT subpopulation of patients with high CVR (SCORE ≥ 5%, no history of CVD). The raw mean calculated CVR changed from 14.3% (A) and 13.4% (B) at baseline to 13.4% (A) and 12.3% (B) at follow-up. Adjusted for baseline the follow-up CVR were 13.1% (95% CI 12.6%-13.6%) (A) and 12.6% (95% CI 12.2%-13.1%) (B) with a group difference (A vs. B) of 0.5% (-0.2%-1.1%), p = 0.179. The group difference was -0.05% in patients of GPs familiar with global CVR and 1.1% in patients of GPs not familiar with with global CVR. However, this effect modification was not significant (p = 0.165). Pooled over groups, the absolute CVR reduction from baseline was 1.0%, p < 0.001. The ICC was 0.026 (p = 0.002).

Sensitivity analysis with EM imputation yielded similar results. Sensitivity analysis with complete cases confirmed these results (-1.0% vs. -1.3%, cf. Table 2).

**Table 2:** **Patients at high risk in primary prevention**

	**Complex intervention A n = 675**		**Simple intervention B n = 649**		**A vs. B**				
	**Mean FU**	**95% CI**	**Mean FU**	**95% CI**	**Difference/Odds ratio**	**95% CI**	**p**	**ICC**	**p**
CVR	13.0%	12.5%-13.6%	12.5%	11.9%-13.1%	Δ = 0.5%	-0.3%-1.3%	0.197	0.029	0.004
RR systolic [mmHg]	138.0	136.4-139.7	137.3	135.6-139.1	Δ = 0.7	-1.7-3.1	0.563	0.082	<0.001
RR diastolic [mmHg]	80.5	79.6-81.4	80.0	79.0-80.9	Δ = 0.5	-0.8-1.8	0.461	0.078	<0.001
Control rate RR < 140/90	46.7%	40.4%-53.1%	46.9%	40.3%-53.5%	OR = 0.99	0.68-1.45	0.966	0.128	<0.001
Control rate RR < 160/95	86.8%	83.1%-90.6%	88.9%	85.4%-92.3%	OR = 0.82	0.51-1.31	0.405	0.141	<0.001
Cholesterol [mg/dl]	221.9	219.1-224.6	220.9	218.1-223.7	Δ = 1.0	-3.0-4.93	0.634	0.015	0.085
Current smoker	12.0%	9.7%-14.4%	14.4%	11.9%-17.0%	OR = 0.71	0.44-1.16	0.171	0.093	0.019
ASS	21.4%	19.1%-23.7%	23.9%	21.0%-26.8%	OR = 0.67	0.37-1.19	0.172	0.206	<0.001
Statins	23.3%	19.7%-26.9%	19.4%	15.8%-23.0%	OR = 1.52	0.89-2.62	0.128	0.206	<0.001

Outcomes at follow up in subpopulation at high CVR (10-year mortality ≥ 5%), but no manifest CVD (complete cases), adjusted for baseline and taking cluster effects into account by use of a mixed model.

Similarly, hypertension control (BP <140/90 mmHg) improved in the same subpopulation from 38.1 to 45.9% in the complex intervention group, and from 35.6 to 46.5% in the simple intervention group, with adjusted follow-up control rates of 46.7% (95% CI 40.4%-53.1%) (A) and 46.9% (95% CI 40.3%-53.5% (B) and an adjusted odds ratio (A vs B) of 0.99 (95% CI 0.68-1.45), p = 0.966 (Table 2). Pooled over groups, the increase was 9.4% (p < 0.001).

In the complex intervention group, the reported smoking rate decreased from 19.4 to 13.5% compared to a reduction in the simple intervention group from 15.6 to 13.6%. The adjusted odds ratio was not significant (Table 2).

In the subpopulation of patients with low CVR mean calculated 10-year (CRV) increased slightly during the intervention period (+0.4% vs. +0.3%), with no significant difference between the 2 groups (Table 3). Changes in hypertension control and smoking rates were small and not significant (data not shown).

**Table 3:** **Patients at low risk in primary prevention**

	**Complex intervention A n = 366**		**Simple intervention B n = 384**		**A vs. B**				
	**Mean FU**	**95% CI**	**Mean FU**	**95% CI**	**Difference/Odds ratio**	**95% CI**	**p**	**ICC**	**p**
CVR	2.9%	2.7%-3.1%	2.7%	2.5%-2.9%	Δ = 0.2%	-0.1%-0.5%	0.282	0.032	0.043
RR systolic [mmHg]	134.0	132.1-135.9	134.3	132.3-136.2	Δ = -0.2	-2.9-2.5	0.866	0.097	<0.001
RR diastolic [mmHg]	80.9	79.9-82.0	80.7	79.7-81.8	Δ = 0.2	-1.3-1.7	0.798	0.063	<0.001
Control rate RR < 140/90	58.1%	51.3%-64.8%	54.0%	47.1%-60.9%	OR = 1.19	0.78-1.82	0.411	0.098	<0.001
Control rate RR < 160/95	90.2%	86.8%-93.6%	90.7%	87.4%-94.0%	OR = 0.94	0.55-1.62	0.830	0.051	0.205
Cholesterol [mg/dl]	216.6	213.0-220.1	219.3	215.7-222.8	Δ = -2.7	-7.7-2.3	0.294	0.021	0.103
Current smoker	16.2%	12.5%-20.0%	16.4%	12.5%-20.3%	OR = 0.98	0.48-1.99	0.948	0.240	<0.001
ASS	9.0%	7.0%-10.9%	9.2%	7.3%-11.2%	OR = 0.89	0.32-2.43	0.815	0.318	0.001
Statins	18.1%	14.6%-21.6%	13.5%	10.2%-16.7%	OR = 1.89	0.98-3.62	0.056	0.150	0.010

Outcomes at follow up in subpopulation at low CVR (10-year mortality < 5%) with no manifest CVD (complete cases), adjusted for baseline and taking cluster effects into account by use of a mixed model.

In the subpopulation of patients with manifest CVD, little or no effects were detected in mean blood pressure, hypertension control and cholesterol level. In the complex intervention group, the smoking rate fell significantly from 20.8 to 15.3% compared to a small change in the control group from 23.3 to 20.8% (Table 4). Neither of these differences in smoking rates was statistically significant.

**Table 4:** **Patients with manifest cardiovascular diseases**

	**Complex intervention A n = 327**		**Simple intervention B n = 279**		**A vs. B**				
	**Mean FU**	**95% CI**	**Mean FU**	**95% CI**	**Difference/Odds ratio**	**95% CI**	**p**	**ICC**	**p**
RR systolic [mmHg]	134.3	132.5-136.1	133.8	131.8-135.7	Δ = 0.5	-2.2-3.2	0.698	0.033	0.080
RR diastolic [mmHg]	78.1	77.0-79.2	79.1	77.9-80.2	Δ = -1.0	-2.5-0.6	0.243	0.048	0.020
Control rate RR < 140/90	59.2%	52.7%-65.7%	56.9%	49.9%-63.8%	OR = 1.11	0.73-1.70	0.632	0.076	0.015
Control rate RR < 160/95	90.2%	86.4%-93.9%	88.9%	84.7%-93.1%	OR = 1.15	0.66-1.99	0.623	0.037	0.280
Cholesterol [mg/dl]	197.8	194.1-201.6	199.8	195.8-203.9	Δ = -2.0	-7.6-3.6	0.482	0.000	1.000
Current smoker	15.2%	11.2%-19.1%	19.2%	15.1%-23.2%	OR = 0.57	0.26-1.26	0.163	0.258	0.005
ASS	73.0%	69.2%-76.7%	73.3%	69.3%-77.2%	OR = 0.96	0.46-2.01	0.917	0.230	<0.001
Statins	64.1%	57.2%-71.0%	48.2%	39.7%-56.7%	OR = 2.64	1.35-5.16	0.005	0.280	<0.001

Outcomes at follow up in subpopulation at high CVR (10-year mortality ≥ 5%), but no manifest CVD (complete cases), adjusted for baseline and taking cluster effects into account by use of a mixed model.

With regard to prescription rates of statins, intervention effects were much higher in the group of patients with manifest CVD (OR = 2.63, p = 0.005; Table 4). In patients with low CVR, significance of a moderate intervention effect was just missed (OR = 1,90, p = 0,053; Table 3), and in patients with high CVR, there was no intervention effect (Table 2). However, the differences between the intervention effects of CVR groups were not significant (p = 0.0639). Pooled over groups, the follow-up statin prescription rates were 30.7% (95% CI 27.2%-34.2%) (A) and 24.1% (95% CI 20.5%-27.7%) (B) with an odds ratio (A vs. B) of 1.9 (1.2-3.1), p = 0.010, ICC = 0.225, p < 0.001. Prescription rates of ASS did not differ between groups (Tables 2, 3 and 4).

ICCs were mostly significant. Lowest in CVR, they were high in medication, control rate and smoking (Tables 2, 3 and 4) in each of the subgroups.

## Discussion

### Main findings

While the targeted subpopulation of patients with high CVR (SCORE ≥ 5%, but no manifest CVD) did show a significant decline of calculated CVR in both groups, our complex (A) intervention offered no additional effects on CVR reduction compared with the simple (B) intervention. There was, however, a large and significant intervention effect on statin prescription rates in patients with manifest CVD.

### Interpretation of the results

The fact that our complex intervention did not have the intended greater effect than the simple (postal) intervention has several possible explanations relating to (i) the design of the interventions, (ii) other aspects of the study design, and (iii) the participating physicians and patients.

First, despite its complexity and strengths, our complex intervention may have been insufficient, especially in view of the deeply rooted beliefs and routines of GPs. Thus it needs to be modified if consideration of global CVR (and subsequently shared decision making in this area) replaces single risk factor management in the minds of doctors and patients.

Second, the observation period could also have been too short for a change in calculated CVR to occur, although we do not believe that the observed effect would have increased with time without further boosting of the intervention.

Third, it may be that patients were reluctant to comply with additional treatment efforts, and/or that the physicians were eventually not convinced by the new concept and unwilling to change their routines. On the level of GPs, familiarity with the concept of global CVR may have affected the intervention effect. Although the interaction test was not significant, the results suggest that the intervention might have been effective in a group of GPs not familiar with the concept of CVR. However, we had not taken into account stratification of GPs according to CVR knowledge for the estimation of the intervention effect, and the power of the study was insufficient to demonstrate an intervention effect in a specific subgroup. Thus, for clarification another trial focusing on this subgroup would be required.

While there was no group difference, both groups showed a significant reduction of CVR. This may have external and/or internal causes: External causes or secular trends include the introduction of disease management programs for coronary heart disease and diabetes mellitus in Germany in 2002, both affecting primary care and setting incentives to improve CVR factor management. Also, the concept of considering global CVR is a subject directed at primary care physicians in some CME programs. Conceivable internal causes comprise: (1) an equally strong effect of both the complex (A) and simple (B) (postal) intervention; (2) an increased awareness for reducing CVR factors in both the simple and complex intervention group (Hawthorne effect) at the level of GPs and patients; (3) a regression to the mean effect of our baseline and follow-up measurements of both blood pressure and cholesterol; and (4) a reporting bias on the side of the physicians participating in the study in the sense of social expectancy. This study yielded no evidence for either of these explanations, but we believe that both the secular trend and an increased unspecific attention on the side of the participating physicians could account for much of the observed effect.

Among the secondary outcomes, we did find a large and significant intervention effect on statin prescription rates in patients with manifest CVD, and a moderate intervention effect that just missed significance in patients with low CVR, but no such effect in patients with high CVR. The increased statin prescription rate in patients with manifest CVR is congruent with our recommendation to rigorously lower CVR in these patients, and thus seems in line with our complex (peer) intervention. Perhaps this particular, straightforward (and familiar) component of the intervention message was received by the GPs most strongly. On the other hand, the (albeit just not significant) moderate intensification of statin treatment in patients with low CVR, and the absence of a group difference in statin prescriptions for patients with high CVR do not add up to a comprehensible effect in the sense of our intervention with regard to evidence-based treatment in primary prevention, nor can we provide an alternative explanation. The significant increase of statin prescriptions in the pooled analysis suggests an increase of general awareness of GPs concerning other cardiovascular risk factors apart from blood pressure.

Finally, intraclass (intracluster) correlations (ICC) are a measure of the variability between patients of different GPs in relation to the variability of all patients. The high ICC values seen for ASS and Statin reflects the dependency introduced by the GP of prescribing it in most patients or not prescribing it in any. However, with considerable variability between GPs in medication rates, hypertension control or smoking, the variability with CVR was very small in both groups. It seems that the GPs of both groups go different ways in treating their patients, but agree in the goal of CVR reduction in patients with high CVR.

### Study findings in the context of other research

Traditionally, trials aimed at better management of arterial hypertension are designed to improve blood pressure control, regardless of global cardiovascular risk [26,28]. There are few papers available on the implementation of the concept of global CVR in general, and of the use of cardiovascular risk tables in particular, in the management of arterial hypertension in the family-practice setting [15,20]. Few intervention studies have addressed hypertension management in general practice that explicitly consider global cardiovascular risk, and to our knowledge ours is the first to be designed to primarily demonstrate a change in calculated CVR.

One study, however, did calculate CVR before and after the intervention as a secondary outcome; it found, like ours, a small positive effect of both a simple and a complex intervention, i.e. patient encouragement to change lifestyles by means of a nurse-led cardiovascular management strategy, but no superiority of the complex intervention [29]. A second study found positive effects of using decision-aids - such as electronic or paper-based CVR calculators facilitating shared decision-making between physicians and patients on patients’ satisfaction and involvement - without negative effects on the calculated CVR [16].

An intervention trial, including the training of physicians in using a guideline and a decision support tool, did not have the intended effect on GP performance, or patients’ self-reported lifestyle and risk-perception [21]. The authors suggest that improvements in the management of global CVR may have positive effects especially in high risk patients who were not included in their study. However, our study that included high risk patients (albeit in a different setting) does not confirm this assumption.

Thus, while there is a strong scientific consensus that the concept of treating cardiovascular risk rather than single risk factors ought to be implemented in primary care, including greater patient involvement and shared decision-making, there is very little understanding of how this could be undertaken and little evidence of successful models.

### Strengths and weaknesses of the study

The prominent strengths of this study include the high number of participating primary care physicians and patients, and the cluster-randomized design that minimizes the risk of selection bias at the level of physicians. The multifaceted and thorough design of the complex peer-intervention is also a strength, even if eventually it proved ineffective.

Our study has also methodological limitations. The selected regions and GPs were chosen for convenience, which means that the results may not be fully representative of all German GPs. Despite our careful design, we cannot exclude a selection bias at the patient level, possibly due to non-adherence to the rules of consecutive patient enrolment on the side of the physicians. Furthermore, there could be a Hawthorne effect, i.e. that taking part in a study can be a relevant intervention itself both at the level of patients and GPs. This is a principle methodological bias of interventions in healthcare research.

Another possible limitation lies in our decision to compare the complex intervention not with usual care, but with a simple intervention as the control group. This is due to our pre-condition that the benefit of the complex, and therefore costly, intervention should only be found relevant if the benefit can be demonstrated against a less complex, cheaper intervention. In many GP settings, however, our simple intervention comes close to usual care, as guidelines are often posted to GPs. Systematic reviews of interventions to change professional practice show that passive dissemination of information has little or no effect on professional behavior [26,30].

Finally, our analysis does not take into account the variability of follow-up-time (6 versus 9 months), and we cannot tell whether duration of follow-up was associated with any of the outcomes. Follow-up at 6 and 9 months occurred only at GP level, not at patient level, so the follow up data sets reflect cumulated, undated changes of medication, BP-levels, cholesterol-levels, smoking status etc. during the observation period of 6 or 9 months. Therefore, an analysis would not yield reliable results.

## Conclusions

Treating patients with arterial hypertension according to their global CVR rather than to set value limits is a necessary change of paradigm in family practice. Few existing intervention trials have been able to demonstrate marked effects, and our study being the first designed to measure the effect of an intervention of physician peer-education on calculated patient CVR is no exception.

We conclude that our approach to implementing the concept of global CVR into primary care was probably not comprehensive and sufficiently sustainable to change deep-rooted traditional risk factor treatment, which is in accordance to the results of our embedded qualitative study [31]. Given that the concept of global CVR implies increased patient participation in treatment decisions, future research should identify outcome parameters that do take into account the informed choice of patients, even if the latter might not lead to a lower CVR.

## Abbreviations

BP: Blood pressure
CVD: Cardiovascular disease
CVR: Cardiovascular risk
EM algorithm: Expectation–maximization algorithm
ESC: European society of cardiology
GP: General practitioner
ITT: Intention to treat
LOCF: Last-observation carried forward
RCT: Randomised controlled study
SCORE: Systematic COronary Risk Evaluation
